# Optimizing the Protection of Cattle against *Escherichia coli* O157:H7 Colonization through Immunization with Different Combinations of H7 Flagellin, Tir, Intimin-531 or EspA

**DOI:** 10.1371/journal.pone.0128391

**Published:** 2015-05-28

**Authors:** Tom N. McNeilly, Mairi C. Mitchell, Alexander Corbishley, Mintu Nath, Hannah Simmonds, Sean P. McAteer, Arvind Mahajan, J. Christopher Low, David G. E. Smith, John F. Huntley, David L. Gally

**Affiliations:** 1 Moredun Research Institute, Edinburgh, United Kingdom; 2 Division of Immunity and Infection, The Roslin Institute, University of Edinburgh, Edinburgh, United Kingdom; 3 Biomathematics and Statistics Scotland, Edinburgh, United Kingdom; 4 Institute of Infection, Immunity & Inflammation, University of Glasgow, Glasgow, United Kingdom; U. S. Salinity Lab, UNITED STATES

## Abstract

Enterohemorrhagic *Escherichia coli* (EHEC) are important human pathogens, causing hemorrhagic colitis and hemolytic uraemic syndrome in humans. *E*. *coli* O157:H7 is the most common serotype associated with EHEC infections worldwide, although other non-O157 serotypes cause life-threatening infections. Cattle are a main reservoir of EHEC and intervention strategies aimed at limiting EHEC excretion from cattle are predicted to lower the risk of human infection. We have previously shown that immunization of calves with recombinant versions of the type III secretion system (T3SS)-associated proteins EspA, intimin and Tir from EHEC O157:H7 significantly reduced shedding of EHEC O157 from experimentally-colonized calves, and that protection could be augmented by the addition of H7 flagellin to the vaccine formulation. The main aim of the present study was to optimize our current EHEC O157 subunit vaccine formulations by identifying the key combinations of these antigens required for protection. A secondary aim was to determine if vaccine-induced antibody responses exhibited cross-reactive potential with antigens from other EHEC serotypes. Immunization with EspA, intimin and Tir resulted in a reduction in mean EHEC O157 shedding following challenge, but not the mean proportion of calves colonized. Removal of Tir resulted in more prolonged shedding compared with all other groups, whereas replacement of Tir with H7 flagellin resulted in the highest levels of protection, both in terms of reducing both mean EHEC O157 shedding and the proportion of colonized calves. Immunization of calves with recombinant EHEC O157 EspA, intimin and Tir resulted in the generation of antibodies capable of cross-reacting with antigens from non-O157 EHEC serotypes, suggesting that immunization with these antigens may provide a degree of cross-protection against other EHEC serotypes. Further studies are now required to test the efficacy of these vaccines in the field, and to formally test the cross-protective potential of the vaccines against other non-O157 EHEC.

## Introduction

Enterohemorrhagic *Escherichia coli* (EHEC) are worldwide zoonotic pathogens which cause gastro-intestinal disease in humans with potentially life-threatening consequences as a result of systemic Shiga toxin (Stx) activity. Ruminants, and cattle in particular, are the major reservoir of EHEC and humans are colonized via direct or indirect contact with ruminant feces [1–4]. Intervention strategies aimed at limiting colonization and shedding of EHEC from cattle are predicted to reduce the incidence of human disease [5,6], and the development of intervention strategies in cattle has received considerable attention over the last decade.

The EHEC serogroup responsible for most human cases in North America and the UK is O157; however other emerging serogroups are a threat to human health and are more prevalent than O157 in some countries [7]. In recognition of the growing importance of non-O157 EHEC serotypes, six non-O157 serogroups (O26, O45, O103, O111, O121, and O145) have recently been classified as adulterants in the USA [8], meaning that if they are detected in meat batches destined for retail sale then these must be withdrawn at considerable cost to the meat processing industry. Despite these costs, there is little financial incentive for cattle producers themselves to implement interventions, as EHEC infections in cattle are largely asymptomatic and there is currently no evidence that these infections are a direct cause of production losses. Furthermore, there are no statutory requirements for producers to control EHEC in their herds. Consequently, to maximise uptake by the livestock industry any intervention in cattle will need to be cost-effective and supported by clear evidence that such treatments reduce the incidence of human infection.

A number of interventions in cattle have been tested to date including vaccination, probiotics, nutritional manipulation, bacteriophage therapy and biosecurity measures [9–12]. A survey of published interventions has identified vaccines that target adherence and iron regulation as the most efficacious to date [11], and two commercially available vaccines exist, both of which are subunit vaccines consisting of native bacterial proteins: the first vaccine is based on siderophore receptor and porin proteins (SRP) which presumably target bacterial iron uptake (Epitopix LLC, Willmar, Minnesota, U.S) [13,14] whereas the second is based on secreted protein preparations containing components of the bacterial type-III secretion system (T3SS) (Econiche, Bioniche Life Sciences Inc., Belleville, Ontario, Canada) [15–17], which is critical for adherence to and colonization of the bovine intestinal epithelium [18,19]. There is, however, considerable variation in how these vaccines perform in the field [20], which may partly reflect issues with the reproducibility of native bacterial protein preparations. Recombinant subunit vaccines based on T3SS proteins have been shown to be effective at limiting *E*. *coli* O157 colonization and shedding in cattle, sheep and goats [21–24], and we have previously shown that targeting both H7 flagella and the T3SS appears be more effective than targeting the T3SS alone [21]. Recombinant technologies have the added benefit of generating reproducible levels of protein at high purify, thus reducing potential batch-to-batch variation during manufacturing.

To further refine current *E*. *coli* O157 cattle vaccines, a number of properties would be an advantage. Firstly, vaccines should have the ability to cross-protect against other non-O157 EHEC serogroups, which does not appear to be the case for SRP-based vaccines [25,26], but has not yet been tested for T3SS-based vaccines. Secondly, vaccine production should be highly reproducible. Additionally, manufacturing costs should be low to allow sales of these vaccines at a price attractive to the livestock sector, with costs of $3 per dose or less being suggested as a commercially viable production cost [27]. For subunit vaccines where the cost of production is linked to the number of components within the vaccine, this means minimising the number of components required for protection.

In our previous studies, we demonstrated that immunization of cattle with a combination of recombinant EspA, Intimin and the translocated intimin receptor (Tir) provided significant protection against subsequent challenge with EHEC O157, and that the addition of purified H7 flagellin enhanced this protective effect [21]. EspA, intimin and Tir are bacterial proteins involved in T3SS mediated attachment to the host epithelium. EspA is the major structural component of the injection filament of the T3SS, which connects to a pore in the host cell membrane composed of EspB/EspD, and allows a number of effector proteins, including Tir, to be injected into the host cell to promote bacterial binding and modulate host innate immune responses [28]. Following injection, Tir inserts into the apical host cell membrane and acts as receptor for intimin present in the outer membrane of the bacterial cell wall, and the Tir-intimin association leads to Arp2/3-directed cytoskeletal re-arrangement and intimate attachment of the bacteria to the host cell [29,30].

As intimin and Tir represent cognate ligand and receptor, it may be possible to simplify our current EspA, Intimin and Tir-based recombinant vaccine by targeting only one of these components without any loss of efficacy. Given that intimin may play an additional role in adherence via interactions with nucleolin and β1-integrin within the host-cell plasma membrane [31], the removal of Tir rather than intimin would be expected to have the least impact on vaccine efficacy. Furthermore, targeting EspA in a vaccine may also interfere with Tir translocation into the host cell, and therefore a vaccine based on EspA and Intimin alone may effectively target Tir-based adherence without the need to include Tir antigen within the vaccine. H7 flagella are thought to be involved in initial anchoring of the bacteria to the host cell [32], and the enhanced protection seen by raising responses to both T3SS- and H7 flagella-mediated attachment is probably a result of the vaccine targeting two different bacterial adherence mechanisms. Therefore, we hypothesised that addition of H7 flagellin to a simplified T3SS-based subunit vaccine (EspA and Intimin alone) could result in an effective but simplified vaccine formulation.

The primary aim of this study was to optimize our current EHEC O157 subunit vaccine formulations based on EspA, intimin, Tir and H7 flagellin [21] by identifying the key combinations of these antigens required for protection. To achieve this we addressed the following two questions: (i) Can Tir be removed from an EspA, intimin and Tir-based vaccine without any loss in efficacy, thus resulting in a simplified T3S-based vaccine containing only EspA and intimin?; (ii) Would inclusion of H7 flagellin augment the protection generated by this simplified T3S based vaccine? Given the relatively high sequence similarities between EspA, Intimin and Tir from different EHEC serotypes (BLASTp), a secondary aim of this study was to determine whether antibodies generated against EHEC O157 versions of these proteins are able to cross-react with antigens from other EHEC serotypes.

## Materials and Methods

### Ethics statement

Immunizations and oral bacterial challenges of cattle were performed at Moredun Research Institute (MRI) under Home Office licence 60/3179. Ethical approval was obtained from the MRI Animal Experiments and Ethical Review Committee.

### Purification of *E*. *coli* O157 proteins

Vaccine antigens were generated as follows: Flagellin (H7) was isolated from *E*. *coli* O157:H7 (Stx−) strain ZAP984, and recombinant 6 x histidine-tagged EspA, intimin-531 and Tir were over-expressed and purified as previously described [21]. Purity of each protein was verified using sodium dodecyl sulphate (SDS)-polyacrylamide gel electrophoresis (PAGE) followed by Simply Blue staining (Invitrogen, San Diego, CA) and single bands of the anticipated molecular weight for each protein were identified (S1 Fig). Protein concentration was assessed using a bicinchoninic protein assay kit (Pierce Biotechnology, Inc., Rockford, IL, USA).

For western blotting, secreted protein preparations and whole cell preparations were generated from the following EHEC strains: *E*. *coli* O111:H21 (ZAP 118), *E*. *coli* O103:H2 (ZAP 305), *E*. *coli* O145 (ZAP 1073), *E*. *coli* O26:H11 (ZAP 1078), *E*. *coli* O26:H- (ZAP 1130), *E*. *coli* O26 (ZAP 1147) and *E*. *coli* O157:H7 (ZAP198). Secreted protein preparations were prepared by culturing the bacterial strains in 50 ml of DMEM-HEPES (Sigma-Aldrich) at 37°C and shaking at 200 rpm, conditions which promote expression of T3SS proteins [33], until an optical density (OD) of ~1.0 was achieved. Bacterial cells were removed by centrifugation at 4000 × *g* for 20 min and supernatants filtered using a 0.45 μm filter. Bovine serum albumin was added to a final concentration of 2 μg/ml and proteins precipitated overnight at 4°C by addition of 10% (w/v) trichloroacetic acid. Following centrifugation at 5000 × *g* for 30 min at 4°C, pelleted proteins were re-suspended in 1.5M Tris-HCl buffer, pH 8.8. The concentration of EspA within the secreted protein preparations was estimated by densitometry of Simply Blue-stained SDS-polyacrylamide gels using a recombinant EspA standard and ImageQuant TL software (GE Healthcare, Chalfont St Giles, UK). Whole cells preparations were generated by culturing the bacterial strains as previously described followed by centrifugation at 16,000 × *g* for 5 min. Pelleted cells were then re-suspended in SDS-PAGE loading buffer (Sigma-Aldrich) prior to use.

### Immunization protocol and oral bacterial challenge

Conventionally reared male Holstein–Friesian calves were immunized on three separate occasions at 3 weekly intervals with one of the following immunization protocols shown in Table 1: (1) Group *E-I-T*; EspA, intimin-531 and Tir plus Quil A adjuvant (Quil-A saponin, Brenntag Biosector, Frederikssund, Denmark), (2) Group *E-I*; EspA and intimin-531 plus Quil A, (3) Group *E-I-H7*; EspA, intimin-531 and H7 flagellin plus Quil A and (4) Control; Quil A alone. Calves were housed in conventional sheds on deep straw throughout the study, and fed on a diet of concentrate pellets (Caledonian Rearing Pellets, Davidsons Animal Feeds, Shotts, UK) and grass hay. At the start of the experiment, eight calves were randomly allocated to each immunization group and then calves were randomly allocated into four pens consisting of two calves per immunization group (henceforth referred to as ‘group’) per pen. Based on the data from our previous studies [21,34] we identified the group sizes such that the experiment had adequate power to test the mean differences in bacterial shedding profiles between the immunization groups. During the course of the experiment two calves from two separate groups were euthanased due to pneumonia. The average age of calves at the time of the first immunization was 13 weeks, with an age range of 11–15 weeks. Fecal samples obtained from each calf prior to immunization were confirmed to be negative for *E*. *coli* O157:H7 by immunomagnetic separation, performed according to the manufacturer’s instructions (Dynabeads anti-*E*. *coli* O157, Invitrogen).

**Table 1:** Summary of immunization protocols.

Group	n	Immunization
***E-I-T***	7	60μg EspA, intimin and Tir plus 5mg Quil A
***E-I***	7	60μg EspA and intimin plus 5mg Quil A
***E-I-H7***	8	60μg EspA, intimin and H7 flagellin plus 5mg Quil A
**Control**	8	5mg Quil A

Seven days after the last immunization, calves were orally challenged with 10^10^ CFU nalidixic acid-resistant *E*. *coli* O157:H7 (Stx−) strain ZAP198 [21] and viable *E*. *coli* O157:H7 bacteria per gram of surface feces (CFU/g feces) were enumerated once daily from day 3 to day 9 post-challenge and then every second day until day 23 post-challenge by plating 100 μl of a 1:10 dilution of feces (equivalent to 0.01 g feces) in triplicate onto sorbitol MacConkey agar plates containing 15 μg/ml nalidixic acid (Oxoid) as previously described [34]. Samples were collected from day 3 post-challenge, as the bacteria excreted before day 3 are most likely reflect flow-through of the challenge inoculum and not colonization of the intestinal tract [19]. Further 10-fold serial dilutions of fecal suspensions were plated out if the number of colonies per plate was too high to enumerate. The limit of detection for this direct plating method was ≥ 100 CFU/g feces. If no colonies were recovered from any of the triplicate plates, fecal samples were submitted to broth enrichment in tryptone soya broth (Oxoid) containing 15 μg/ml nalidixic acid overnight at 37⁰C and samples were assigned as positive or negative for broth enrichment. Serum samples were collected 2 days prior to first immunization and 1 day prior to oral bacterial challenge. Bacteriology data is available in the S1 Dataset.

### Quantification of antigen-specific antibody responses

Levels of antigen-specific IgA, IgG1 and IgG2 were determined in serum samples by indirect ELISA as previously described using serial dilutions of serum [21]. Antibody titres were calculated as follows: the OD was plotted against the sample dilution and regression of OD on dilution (on logarithmic scale) allowed the calculation of the endpoint titre with an OD of 0.1 above the average negative control value. Inter-plate variation was normalised to a known positive control sample. Serum antibody data is available in the S2 Dataset.

### Western blot analysis of different EHEC serotypes

Western blotting to investigate IgG1 antibody reactivity was performed on whole cell and secreted protein preparations derived from different EHEC serotypes using pre- and post-immunization serum samples from three individual calves from the *E-I-T* immunization group as previously described [21]. For secreted protein preparations, concentrations were adjusted to give a final loading of 2 μg of EspA per lane. For whole cell preparations, ~10 μg of total protein per lane was used.

### Statistical analyses

#### Bacteriological data analysis

To investigate the effect of immunization group on the mean bacterial count, data were analysed using three different approaches. Initially, to evaluate the difference between total bacterial shedding across the entire experimental period for calves in different immunization groups, the area under curve (AUC) of the mean of the triplicate measures of CFU/g feces for each calf sample was calculated using the trapezoid method. This AUC data (adding a constant value of 1) were subsequently transformed using logarithmic transformation. A linear mixed model (LMM) was fitted to the transformed AUC data incorporating immunization group as a fixed effect and pen as a random effect.

Secondly, if there was at least one non-zero observation from the direct plating method, or one positive observation from the broth enrichment method, the calf sample at that time was defined to be bacterial positive (i.e. the calf was colonized). This binary data, indicating presence of bacteria, was assumed to follow a Bernoulli distribution, and modelled using a generalised linear mixed model (GLMM) in which the logit of the mean proportion of presence of bacteria was a linear predictor comprising both fixed and random effects (Bernoulli GLMM), a form of mixed effect logistic regression.

Finally, the triplicate observations on CFU/g, for bacteria positive samples only, were assumed to follow a Poisson distribution, and modelled using a GLMM in which the logarithm of the mean CFU/g feces was modelled by a linear predictor comprising both fixed and random effects and the logarithm of the corresponding dilution factor was included as an offset variable (Poisson GLMM).

The fixed effects for both Bernoulli and Poisson GLMMs included immunization group, day (relative to challenge) and the interaction between group and day. The fixed effect of day was included as a deviation from the mean day (11.5) and fitted as a linear continuous variable. The GLMMs also included random effects for pen, the interaction between pen and day (fitted as a factor) and calf. For the Poisson GLMM, additionally, the interaction between calf and day was included as a random effect and the dispersion parameter was estimated to account for the overdispersion in the data.

#### Antibody data analysis

The data on log-transformed (base 10) antibody responses were analysed by appropriate linear mixed models in two ways. Firstly, to find the effect of different immunization groups on antibody responses post-immunization (i.e. 1 day prior to oral bacterial challenge), a linear mixed model (LMM) was fitted to post-immunization titre data for each antibody. The model included pen as a random effect and group as a fixed effect. Secondly, the mean difference between pre- and post-immunization titres for each calf was investigated using an LMM incorporating pen and animal within pen as random effects. The model also included group, immunization status (pre or post) and their interaction as fixed effects. To compare the immunization status (post versus pre) within each group, estimated probabilities were adjusted using the False Discovery Rate (FDR) approach as described below.

#### General modelling strategy

Parameters of the LMM were estimated using the residual maximum likelihood (REML) method. For both LMM and GLMMs, overall statistical significance of a fixed effect was assessed from the corresponding *p*-value estimated from the *F*-statistic. If the *F* test was statistically significant (*p* < 0.05) for a fixed effect, comparisons of differences in mean values for levels of a fixed effect were obtained using two-sided probabilities for each comparison. These probabilities were then adjusted using a False Discovery Rate (FDR) approach [35] to take into account the multiple comparisons of means of a factor so that the expected proportion of false positives among all positives (i.e. rejecting the null hypothesis) was less than 5%. The FDR value, denoted in this paper as ‘*p*_*f*_’, therefore summarises the strength of evidence for there being a real difference in a way analogous to a standard *p*-value. Pertinent estimates from the models are given in the text together with standard errors of the estimates.

All statistical analyses were carried out using R software version 3.0.1 with appropriate R packages (stats, MESS, lme4, multcomp, lattice, ggplot2) [36].

## Results

### Effect of immunization on subsequent *E*. *coli* O157:H7 colonization

**Fig 1:**
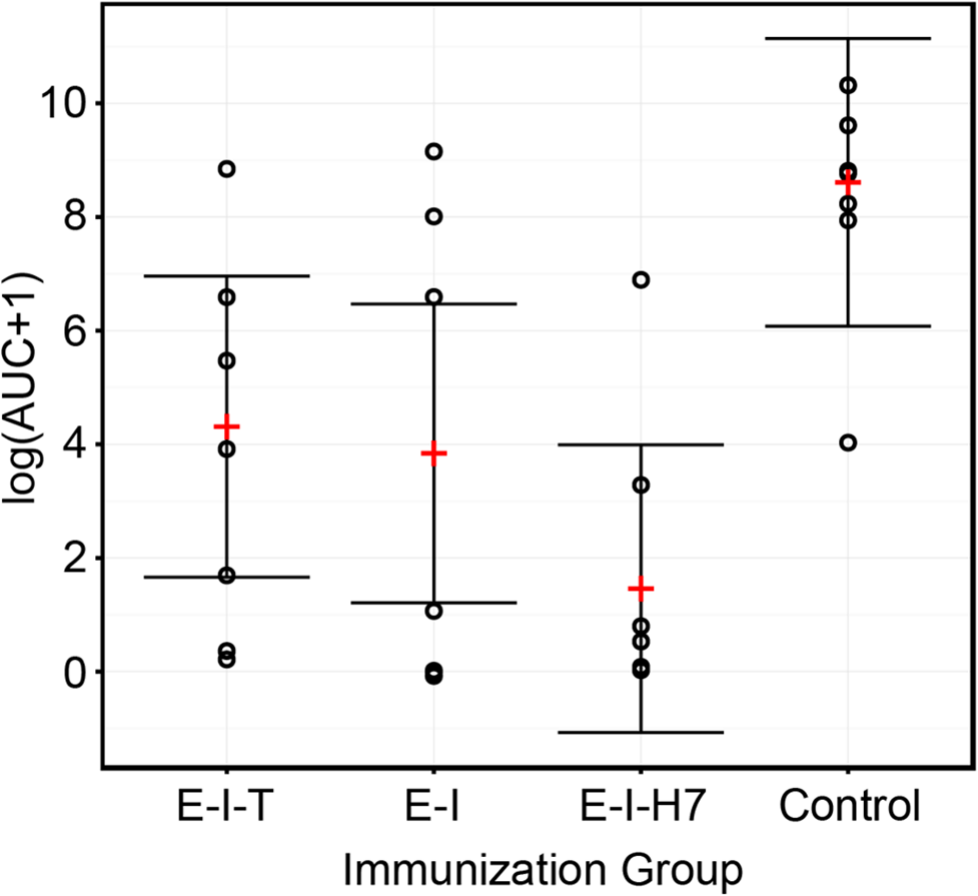
Total shedding of *E*. *coli* O157:H7 following oral challenge of immunized calves. Four groups of calves were immunized intramuscularly with either EspA, intimin and Tir (*E-I-T*), EspA and intimin (*E-I*), EspA, intimin and H7 flagellin (*E-I-H7*) or adjuvant alone (Control) and subsequently orally challenged with *E*. *coli* O157:H7. Faecal bacterial shedding was monitored for 23 days post-challenge and the area under the bacterial shedding curve (AUC) was calculated over the total challenge period for each calf to give an estimate of total bacterial shedding. The AUC data were subsequently transformed using natural logarithmic scale. The plot shows the observed AUC data (open circles) and the estimated mean (plus) and 95% confidence interval (vertical lines) for each immunization group obtained from the linear mixed model fitted to the AUC data.

To estimate total bacterial shedding over the course of the experiment, the AUC was calculated for each individual calf. Fig 1 shows the mean AUC on the log transformed scale and 95% confidence interval for each immunization group estimated from the LMM. Group had a statistically significant (*p* < 0.001) effect on the mean AUC. All three vaccinated groups had statistically significantly (*p*_*f*_ < 0.01) smaller mean AUC (*E-I-T*: 4.31±1.35; *E-I*: 3.84±1.34 and *E-I-H7*: 1.46±1.29 on the log scale) compared with the control group (8.61±1.29). Results showed that the mean AUC of the *E-I-H7* group was marginally smaller than that of the *E-I-T* group (*p*_*f*_ = 0.056).

**Fig 2:**
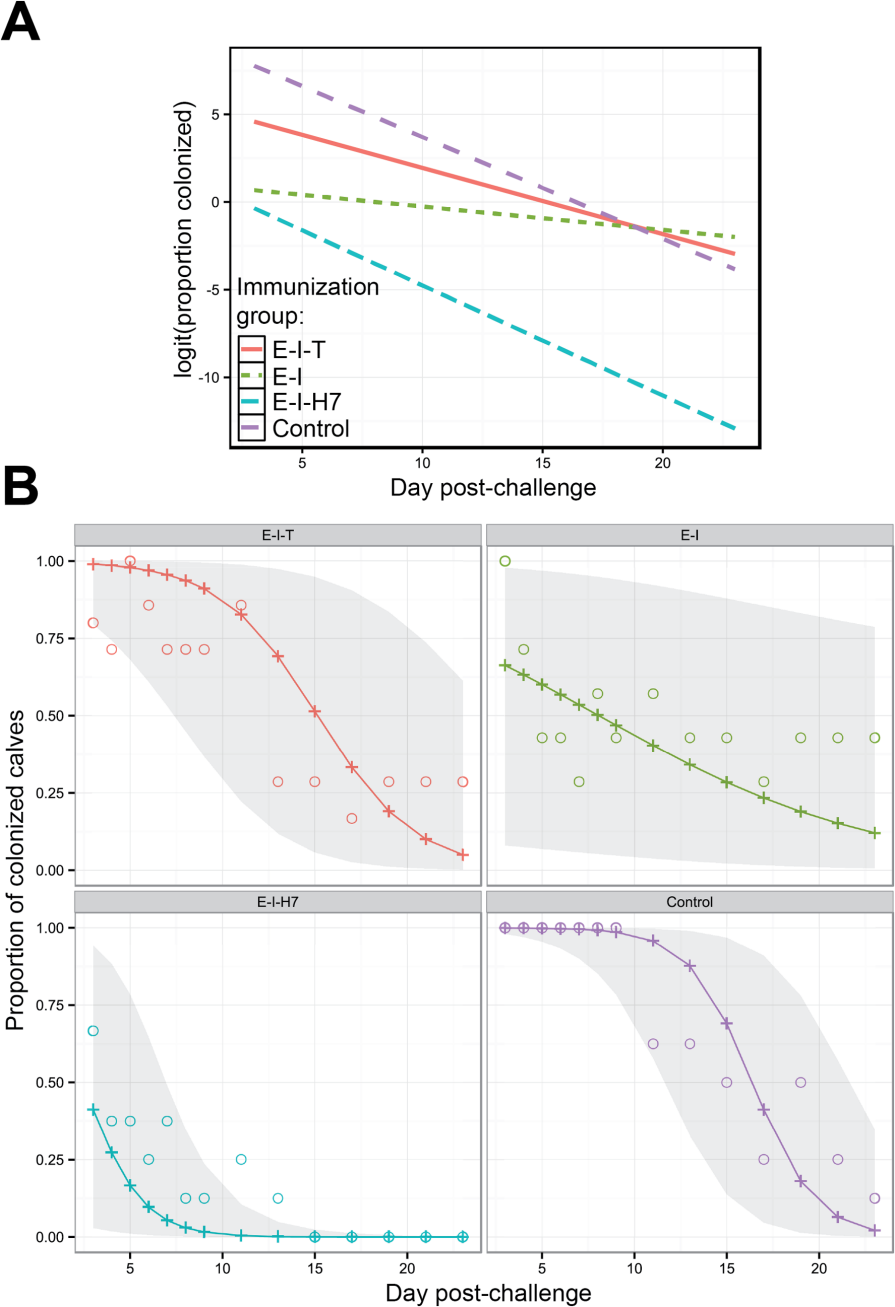
Estimated mean proportions of colonized calves following oral challenge of immunized calves with *E*. *coli* O157:H7. (A) Estimates of linear predictor (logit of proportion of colonized calves) for each immunized group at each day post-challenge. (B) Observed mean proportions (open circles), the estimated mean proportions (plus symbols joined by line) and 95% confidence intervals envelop (shaded region) of colonized calves for each immunized group at each day post-challenge.

The Bernoulli GLMM suggested that the trend over time in the mean proportion of colonized calves following vaccination and subsequent EHEC O157 challenge varied significantly with immunization group (group by day interaction, *p* = 0.002). The estimated mean proportions of colonized calves on the logit scale for each group at each time are summarized in Fig 2A, and the same estimates on the observed scale (i.e. estimated proportions) along with 95% confidence intervals as well as observed mean proportions for each group at each day are presented in Fig 2B.

Estimates of the slopes for each group were negative suggesting that the mean proportion of colonized calves declined with day post-challenge for all groups (Fig 2A). All estimates (on the logit scale) of group-specific slopes were statistically significantly different from zero (*p* < 0.01) except for the *E-I* group (*p* = 0.067) which showed a statistically non-significant rate of decline with an estimate of -0.13±0.07. Comparison of slopes (on the logit scale) between *E-I-H7* (-0.63±0.19), *E-I-T* (-0.38±0.09) and control (-0.58±0.12) groups were not statistically significant (*p*_*f*_ > 0.05) suggesting that the rate of decline in the mean proportion of colonized calves did not differ between these groups (Fig 2A).

Estimated mean proportions of colonized calves for each group at the start of faecal monitoring (i.e. day 3 post-challenge) were compared (Fig 2A and 2B). The lowest estimated means (on the logit scale) of colonized calves at day 3 post-challenge were observed in the *E-I-H7* (-0.67±1.57) and *E-I* (0.61±1.58) groups, and these estimates were statistically significantly (*p*_*f*_ = 0.005 and 0.015, respectively) smaller than that for the control (7.48±1.93) group. The estimated mean proportion (on the logit scale) for the *E-I-T* (4.40±1.63) at day 3 post-challenge was statistically significantly higher than the *E-I-H7* group (*p*_*f*_ = 0.044), but it was not significantly different from that of the *E-I* (*p*_*f*_ = 0.132) and control groups (*p*_*f*_ = 0.258). At the mid-point of the experiment (approximately day 11 post-challenge), the estimated mean proportion of colonized calves in the *E-I-H7* group was less than 0.5% while it was approximately 40%, 83% and 96% for the *E-I*, *E-I-T* and control groups, respectively. At the end of the experiment (day 23 post-challenge), the estimated mean proportion of colonized calves for the *E-I-H7*, *E-I-T* and control groups were 0.0002%, 5% and 2% respectively, but the shedding incidence was still higher for the *E-I* group (12%) (Fig 2B).

**Fig 3:**
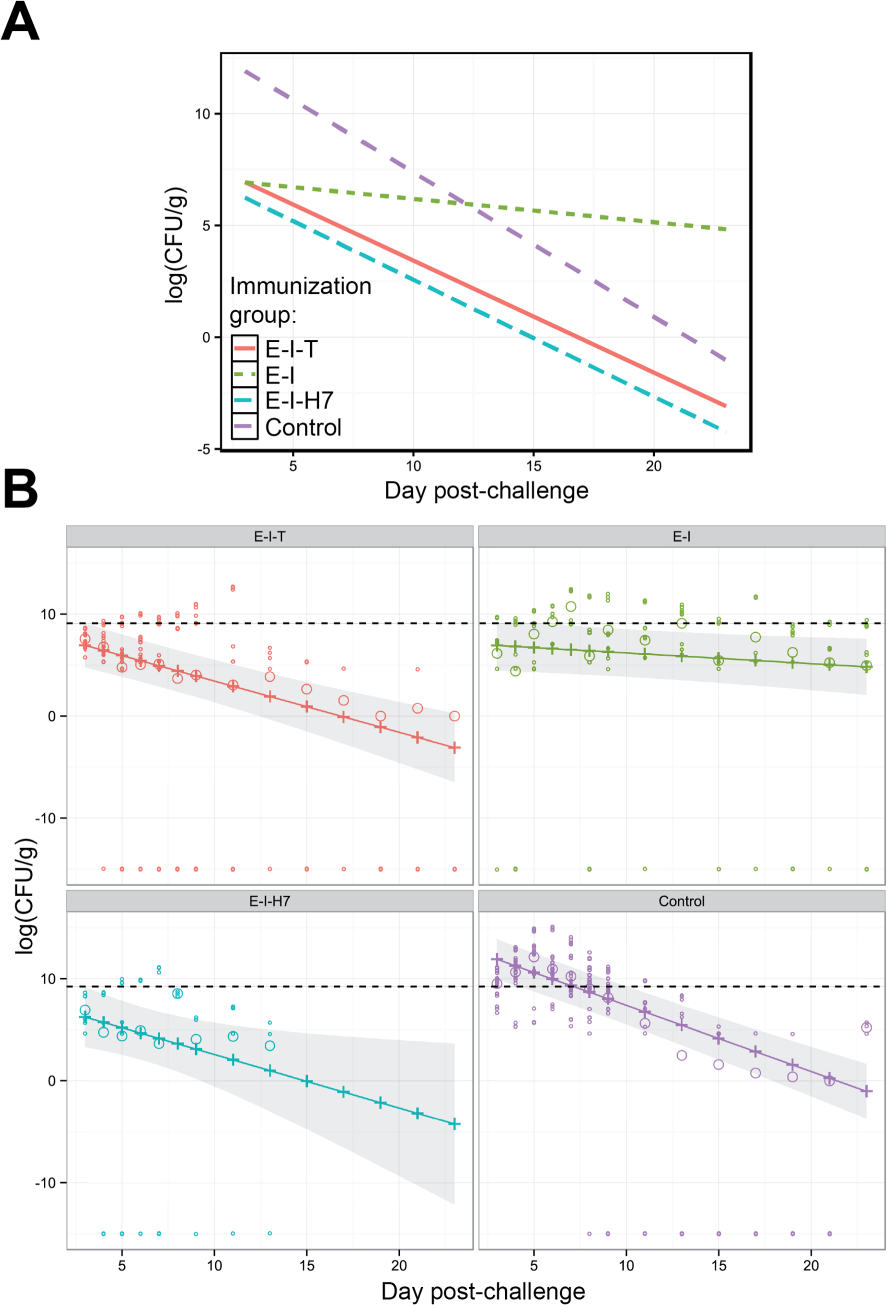
Estimated mean faecal bacterial shedding following oral challenge of immunized calves with *E*. *coli* O157:H7. Graphs represent the triplicate fecal bacterial count data from colonized calf samples only, defined by having at least one non-zero observation from the direct plating method, or one positive observation from the broth enrichment method. (A) Estimates of linear predictor (logarithm of CFU/g feces) for each immunized group at each day post-challenge. (B) Observed mean counts (open circles) (logarithm of CFU/g feces), the estimated mean counts (plus symbols joined by line) and 95% confidence intervals envelop (shaded region) for each immunized group at each day post-challenge. All observed means with zero CFU/g feces count are presented as -15 on the logarithmic scale. Broken horizontal dash line shows the desirable CFU/g feces threshold of 10^4^ (or 9.21 on log scale) CFU/g, below which is predicted to reduce the basic reproductive ratio below 1.0 [37].

The Poisson GLMM suggested that the trend over time in the mean CFU/g feces from colonized calf samples, following vaccination and subsequent EHEC O157 challenge, varied significantly with immunization group (group by day interaction, *p* < 0.001). The estimated mean CFU/g feces of the colonized calves on the log scale for each group at each time are summarized in Fig 3A, and these estimates along with 95% confidence interval as well as observed means (on the log scale) for each group at each day are presented in Fig 3B.

Estimates of the slopes for each group were negative suggesting that the mean CFU count decreased over time for all groups (Fig 3A). Estimates (on the log scale) of slopes for the *E-I-H7* (-0.52±0.23), *E-I-T* (-0.50±0.09) and control (-0.65±0.07) groups were statistically significantly different from zero (*p* < 0.05) while estimates of slope for the *E-I* (-0.10±0.06) group was not statistically significant from zero (*p* = 0.071). The higher estimates of uncertainties at later time points in the *E-I-H7* group, which are reflected by wider confidence intervals (Fig 3B), can be attributed to the fact that no positively colonized animals were present in this group from 15 days post-challenge. Comparisons of slopes (on the log scale) between *E-I-H7*, *E-I-T* and control groups were not statistically significantly different (*p*_*f*_ > 0.05), suggesting that the rate of decline in the log mean CFU count of colonized calves did not differ between these groups (Fig 3A).

Estimated mean CFU counts (on the log scale) of colonized calves for each group at day 3 post-challenge were compared (Fig 3A and 3B). Smaller estimated mean CFU/g feces (on the log scale) of colonized calves at day 3 post-challenge were observed in all immunized groups; *E-I-H7* (5.98±1.45), *E-I-T* (6.69±1.09) and *E-I* (6.87±1.25); and these estimates were statistically significantly (*p*_*f*_ < 0.01) smaller compared with the control (11.58±0.99) group. The mean CFU/g feces counts at day 3 post-challenge were not significantly different (*p*_*f*_ > 0.05) between three immunized groups. At the mid-point of the experiment (approximately day 11 post-challenge), the estimated mean CFU/g feces was approximately 8 and 19 for the *E-I-H7* and *E-I-T* groups, respectively, while it was considerably higher for the *E-I* (>439) and control (>838) groups (Fig 3A). In fact, the mean CFU/g feces for the *E-I* group were higher than other vaccinated groups across all time points, and it continued to shed until day 23 post-challenge even when the mean shedding of the control group had decreased substantially (Fig 3A and 3B). Assuming the mean CFU/g feces threshold value of less than 10^4^ (or 9.21 on log scale) as a desirable threshold [37], it was observed that the 95% confidence intervals for log mean bacterial shedding of *E-I-T* and *E-I-H7* did not exceed the threshold value for all observed time points, while the confidence intervals of the *E-I* group did not include the threshold from day 4 post-challenge. For the control group, 95% confidence intervals for the log mean CFU/g feces did not include the threshold value from day 11 post-challenge (Fig 3B).

### Serum antigen-specific antibody responses

**Fig 4:**
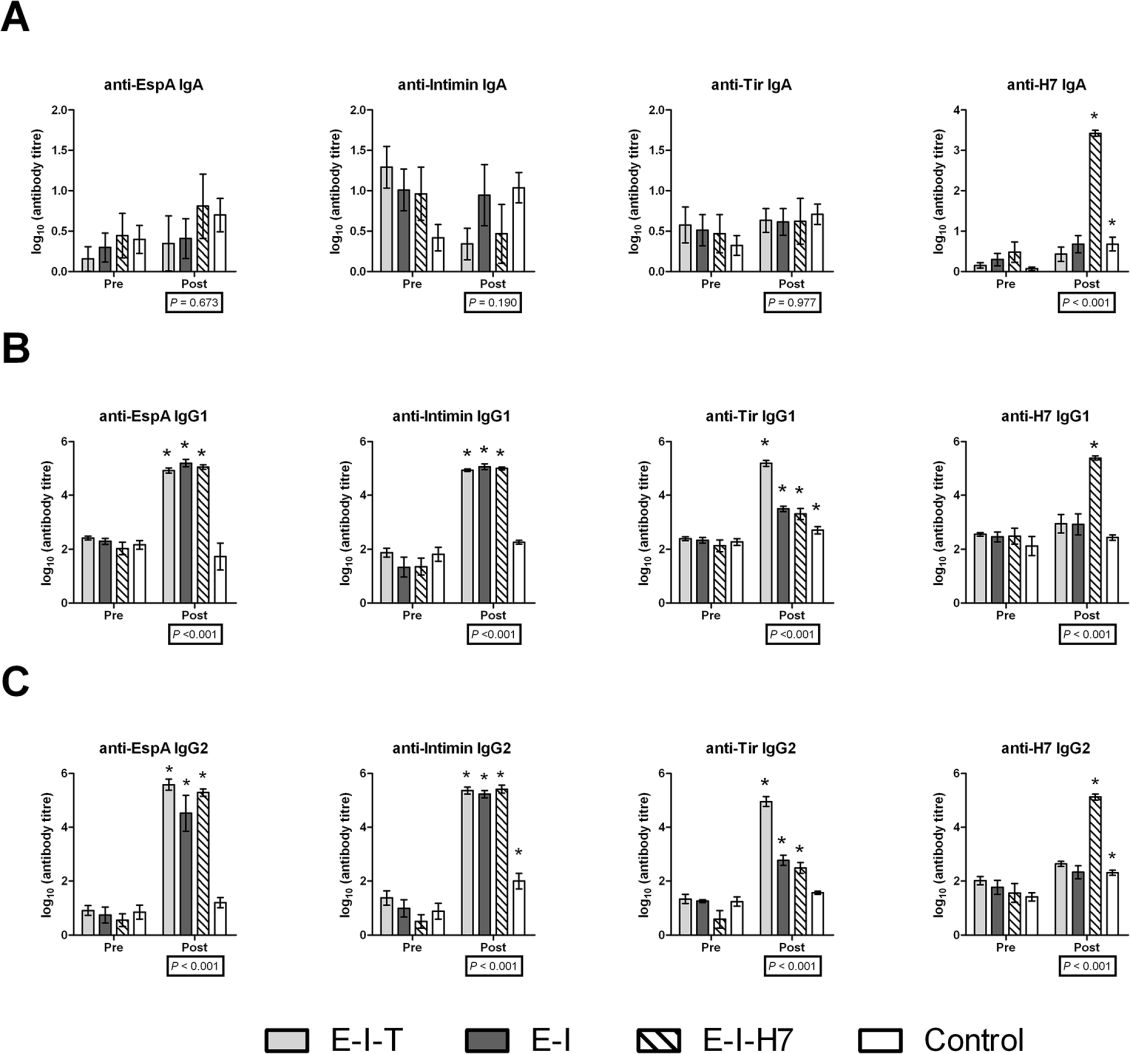
Serum antibody titres of different immunization groups. Mean titres of (A) IgA, (B) IgG1 and (C) IgG2 of EspA-, Intimin-, Tir- and H7 flagellin (H7)-specific antibodies within the serum of calves 2 days prior to the first immunization (Pre) and 6 days after the final immunization (Post) with either EspA, intimin and Tir (*E-I-T*), EspA and intimin (*E-I*), EspA, intimin and H7 flagellin (*E-I-H7*), or adjuvant alone (Control). Each bar represents the mean (on log_10_ scale) and corresponding error bar shows the standard error of mean. Overall statistical significance of immunization group for each antibody titre post-immunization was assessed using a linear mixed model and indicated by a *p*-value below each graph. The mean difference in titres between pre- and post-immunization for each immunization group was investigated using a linear mixed model, and statistically significant (adjusted for false discovery rate, *p*_*f*_ < 0.05) changes are indicated by an asterisk (*).

Serum antibody responses are shown in Fig 4. The effect of immunization group was statistically significant (*p* < 0.001) for all post-immunization antibody titres except for anti-EspA, anti-Intimin and anti-Tir antibodies belonging to the IgA isotype. Paired comparisons of antibody responses to the antigens (pre and post immunization) showed a statistically significant (*p*_*f*_ < 0.05) increase in serum anti-EspA and anti-Intimin IgG1 and IgG2, but not IgA, in calves in all three immunization groups. A significant increase in serum anti-H7 IgA, IgG1 and IgG2 was observed in *E-I-H7* group but not the other two immunization groups, whereas a significant increase in anti-Tir IgG1 and IgG2 was evident in all three immunization groups, although the mean anti-Tir antibody response was greater than two orders of magnitude higher in the *E-I-T* group. Interestingly, a small but significant increase in anti-Intimin IgG2, anti-Tir IgG1 and IgG2, and anti-H7 IgA and IgG2 was observed in the control group, suggesting that calves were naturally exposed to these or similar antigens throughout the course of the study, as it is known that *E*. *coli* strains other than EHEC O157 which are capable of expressing these antigens can be found relatively frequently in bovine feces [38,39].

### Cross-reactivity of immunization-induced antibodies with different EHEC serotypes

**Fig 5:**
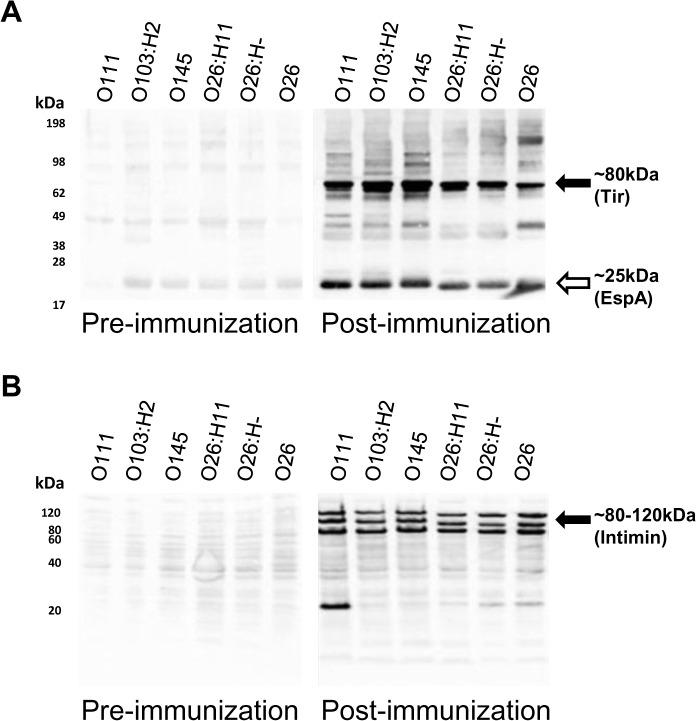
Cross-reactivity of vaccine-induced antibodies with secreted and whole cell antigen preparations from non-O157 EHEC serotypes. Representative western blot of (A) secreted protein preparations and (B) whole cell preparations from EHEC serotypes O111, O103:H2, O145, O26:H11, O26:H- and O26 with serum collected from a calf collected 2 days prior to first immunization (Pre-immunization) and 6 days after the final immunization (Post-immunization) with recombinant EspA, intimin and Tir. Bound IgG1 was visualized by chemiluminescence. The putative identities of EspA, Tir and intimin-reactive bands are indicated in brackets.

To determine whether antibodies generated following immunization with recombinant *E*. *coli* O157:H7 EspA, intimin and Tir were capable of recognising antigens from different EHEC serotypes, western blotting was performed using pre- and post-immunization serum from three individual calves in the *E-I-T* immunized group on secreted protein preparations and whole cell preparations from six different EHEC isolates representing four different serotypes: *E*. *coli* O111:H21, *E*. *coli* O103:H2, *E*. *coli* O145, three *E*. *coli* O26 strains. We focused on IgG1 as antibodies of this isotype were induced to all of the immunizing antigens and it is a prominent isotype at the bovine intestinal mucosa [40], the site of colonization of EHEC [41]. Representative western blots using serum samples from one of the calves are shown in Fig 5, and the results were consistent using serum samples from all three calves tested. Serum from *E-I-T* immunized calves contained IgG1 which reacted with two protein bands in the different supernatant preparations of all the strains tested (Fig 5A). The migration of the recognised proteins was consistent with Tir (~80 kDa) and EspA (~25kDa) based on our own work and other published studies [33,42]. Immunization also resulted in the generation of IgG1 which reacted with three distinct bands of between ~80 to 120 kDa in all whole cell preparations tested. This is consistent with recognition of intimin and possible assembly or degradation variants that do not posses specific C-type lectin and immunoglobulin domains (Fig 5B) [43].

## Discussion

In the present study we compare the protective effect of immunization of cattle with three different combinations of EspA, intimin-531, Tir and H7 flagellin on subsequent excretion of EHEC O157 following experimental oral challenge with the bacteria. All three vaccine formulations (EspA and Intimin [*E-I*], EspA, Intimin and Tir [*E-I-T*], and EspA, Intimin and H7 flagellin [*E-I-H7*]) resulted in a significant reduction in total bacterial shedding following challenge compared to calves administered with adjuvant alone, with mean total shedding being lowest in the group immunized with *E-I-H7* and similar in *E-I* and *E-I-T* immunized groups.

At day 3 post-challenge, when bacteria in feces reflect initial intestinal colonization rather than flow through of the challenge strain [19], there was a similar reduction in group mean faecal shedding in all three immunization groups compared with control, but a reduction in the proportion of colonized calves was only evident in the *E-I* and the *E-I-H7* vaccinated groups, the magnitude of which was greater in the *E-I-H7* group. Strikingly, the rate of decline in both the proportion of calves colonized and the mean bacterial shedding was similar in the *E-I-T*, *E-I-H7* and control groups, but was significantly less for calves immunized with *E-I*, suggesting that immunization with EspA and Intimin alone, whist capable of inducing some protective effect, may also delay clearance of EHEC O157 from the intestinal tract. The reason for this delayed bacterial clearance in the *E-I* group compared to controls is unclear, but could reflect reduced immune activation by the bacteria as a result of reduced colonization levels. Despite lower colonization levels in the *E-I-T* and *E-I-H7* groups, similar rates of bacterial clearance in the other three groups suggest that vaccine-induced immune responses to Tir and H7 may compensate for reduced immune activation by the bacteria, although further studies would be required to verify this. These results also suggest that the protection afforded by these vaccines is primarily due to limiting initial colonization of the intestinal mucosa, rather than increasing the rate of bacterial clearance from the colonized intestinal tract.

Another important observation is that inclusion of Tir or H7 flagellin limited bacterial shedding <10^4^ CFU/g feces at all time-points, which is predicted to reduce the basic reproductive ratio (*R*_0_) to below 1 [37], meaning that one infected individual would infect less than one other individual in an otherwise uninfected population. This would, in theory, prevent maintenance of EHEC O157 infections within groups of cattle where animal-to-animal transmission is the main route of transmission. This restriction of shedding below 10^4^ CFU/g feces did not occur in the control group, indicating that vaccination with *E-I-T* or *E-I-H7* may have a significant impact on EHEC O157 carriage in cattle through reducing cattle-to-cattle transmissions, even though vaccination may have little impact on the rate of clearance of the bacteria in infected individuals.

Thus, immunization with a combination of EspA and Intimin provides a degree of protection against experimental EHEC O157 challenge by limiting initial colonization; however, this formulation appears to delay bacterial clearance from the intestine. Inclusion of either Tir or H7 flagellin in the vaccine formulation augments this protection by restoring a similar rate of bacterial clearance to that seen in the control group and, in the case of H7 flagellin, additionally reducing the mean proportion of calves colonized following challenge. Whilst simplifying the vaccine formulation to EspA and intimin alone may have economic benefits with regard to reduced manufacturing costs, this may come at an unacceptable loss in efficacy. The loss of efficacy with the simplified formulation is also consistent with the lack of protection observed when calves were immunized with EspA alone, despite inducing robust antibody responses [44].

The additional protective effect of Tir inclusion indicates a benefit of targeting both this receptor and its cognate ligand intimin, and this is the first clear demonstration that targeting both Tir and intimin in a vaccine results in improved protection. This may simply be due to more efficient targeting of both components of this interaction by vaccine-induced antibodies. However, unlike the other vaccine antigens tested, Tir is injected into the host epithelial cell, and therefore, it is possible that this protein could be targeted by cytotoxic T cells following presentation of Tir peptides by major histocompatibility complex class I (MHC class I) molecules. Recent evidence indicates that airway epithelial cells are highly efficient at presenting intracellular mycobacterial antigens to CD8^+^ T cells via MHC class I [45], and a similar mechanism may occur in other epithelial cell types including intestinal epithelial cells. Therefore, the additional protection conferred by Tir may in part be related to cytotoxic T cell-mediated killing of colonized epithelial cells. Whilst there is currently no direct evidence that this occurs, EHEC O157 is capable of suppressing cell-mediated immune responses in cattle by targeting of lymphocytes, including CD8α^+^ T cells, via Shiga toxin [46–48]. Furthermore, clearance of EHEC O157 is associated with an up-regulation of Th-1 associated transcripts within the rectal mucosa, the principle site of colonization [41,49], suggesting that a cellular component of the adaptive immune response may be important in EHEC O157 control. Further work is required to define the nature of cell-mediated immunity generated by EHEC O157 vaccines, the bacterial antigens presented by MHC class I in colonized epithelial cells and their contribution to protection.

The inclusion of H7 flagellin resulted in a greater enhancement of protection compared to the inclusion of Tir, and this enhancement was largely related to a reduction in the proportion of calves that became colonized following challenge. This effect has been previously observed following immunization with H7 flagellin alone [34], and is consistent with a proposed role of H7 flagella in initial anchoring of the bacteria to the intestinal epithelium, which allows subsequent colonization of the bovine gut [32]. This suggests that targeting two different adherence mechanisms, i.e. both T3SS and H7 flagellin mediated attachment, is a more effective approach for vaccination against EHEC O157, and is supportive of our previous work which demonstrated that the addition of H7 flagellin enhanced protection achieved by immunization with a combination EspA, Intimin and Tir [21]. As an extension to this study, it would be interesting to determine what level of protection could be achieved through immunization with a combination of a single T3SS protein (e.g. EspA or Intimin) plus H7 flagellin, as this may represent a strategy to further simplify the vaccine formulation without any compromise in efficacy.

A key aim of the study was to define a simple recombinant vaccine formulation which would be both efficacious and cheap to produce. Increasing the number of recombinant proteins would increase manufacturing costs by requiring multiple fermentations and increased quality control measures. However, it is apparent both from this study and from studies where calves were immunized with EspA or H7 flagellin alone [34,44] that reducing the number of recombinant EHEC O157 antigens can result in a reduction in vaccine efficacy, and that a balance between the number of antigen targets and vaccine efficacy exists. In this respect, vaccines based on T3-secreted protein preparations are probably effective as they will contain multiple factors (T3-secreted proteins, LPS, flagellin) which contribute to protection. However, a major drawback to this approach is the lack of reproducibility of supernatant preparations, with potential variation in the levels of both protective antigens within the preparations and other bacterial factors such as LPS, which could influence both vaccine efficacy and safety. The benefit of the recombinant approach is that the vaccine can be manufactured in a highly controlled manner so that the concentration of each protective component is consistent between batches. It could also be possible to reduce the number of fermentations, and thus costs, associated with manufacture of a multiple recombinant antigen vaccine by generating chimeric proteins containing multiple EHEC O157 antigen targets, an approach which has recently been successfully demonstrated in goats [24].

A second key aim of this study was to determine whether antibodies generated following vaccination with recombinant EHEC O157 antigens cross-reacted with antigens from other EHEC serotypes, thus providing some evidence of cross-protective potential against other EHEC serotypes. This indeed appeared to be the case, with IgG1 generated following immunization with recombinant EHEC O157 EspA, Intimin and Tir recognising molecules of appropriate molecular weights within secreted protein and whole cell preparations of EHEC O111, O103, O145 and O26. This result probably reflects the relatively high sequence homology of these proteins between different EHEC serotypes (BLASTp): intimin (82–89% identity), EspA (81–85% identity), Tir (61–66% identity), and is consistent with a previous study where antibodies generated against secreted protein preparations from non-O157 EHEC serotypes cross-reacted with recombinant EspA and Tir from EHEC O157, indicating the presence of shared epitopes [50]. However, caution should be taken in the interpretation of these results: while passive transfer experiments of anti-intimin antibodies in pigs and sheep suggest that antibodies play a key role in vaccine-induced protection against EHEC O157 [51,52], antibody cross-reactivity *per se* may not necessarily equate to protective function. Indeed, a previous study demonstrated that the cross-reactive potential of antibodies generated against T3-secreted protein preparations from different EHEC serotypes did not necessarily correlate with the ability of the antibodies to block bacterial epithelial binding *in vitro* [53]. For EspA, recognition may differ between the functional surface structure and that presented in Western blots, as previous studies have shown that polyclonal antibodies raised against EspA from one serotype appear specific in terms of presented EspA filaments [54]. Thus, while the generation of cross-reactive antibodies by our recombinant EHEC O157 vaccine is promising, the functionality still needs to be formally tested in controlled vaccine efficacy studies using other EHEC serotypes.

## Conclusions

Systemic immunization of calves with a combination of EspA, intimin-531, and H7 flagellin resulted in a significant reduction in shedding of EHEC O157 to levels predicted to significantly impact on EHEC O157 cattle-to-cattle transmission. Vaccine efficacy was reduced if H7 flagellin was replaced by Tir, and further reduced if calves were immunized with EspA and intimin-531 alone. Furthermore, immunization with EspA and intimin-531 alone resulted in delayed clearance of the bacteria from the intestine compared to unvaccinated calves, suggesting that this formulation will not be effective in the field. These results clearly show the benefit of including either Tir or H7 flagellin into an intimin-EspA based vaccine, and also provide further evidence that the combined targeting of both H7 flagella and the T3SS appears to be a more effective vaccine approach compared to targeting the T3SS alone. We also present novel data showing that immunization with EspA, intimin and Tir from EHEC O157 resulted in the generation of antibodies capable of cross-reacting with antigens from other EHEC serotypes, suggesting that immunization with these antigens may provide some degree of cross-protection against other EHEC serotypes. Further studies are now required to test the efficacy of these vaccines in the field, and to formally test the cross-protective potential of these vaccines against other non-O157 EHEC.

## Supporting Information

S1 Dataset Bacteriology data. (XLSX)

S2 Dataset Serum antibody data. (XLSX)

S1 Fig Vaccine antigen preparations. Coomassie blue-stained polyacrylamide gel of *E*. *coli* O157 antigens used in immunizations.
(TIF)

S1 File Completed ARRIVE Guidelines Checklist. (PDF)
