# Supplementary figures and images for: Optimizing the Protection of Cattle against Escherichia coli O157:H7 Colonization through Immunization with Different Combinations of H7 Flagellin, Tir, Intimin-531 or EspA
Source: PLoS One. 2015 May 28;10(5):e0128391. doi: 10.1371/journal.pone.0128391 (PMC4447243; doi:10.1371/journal.pone.0128391)

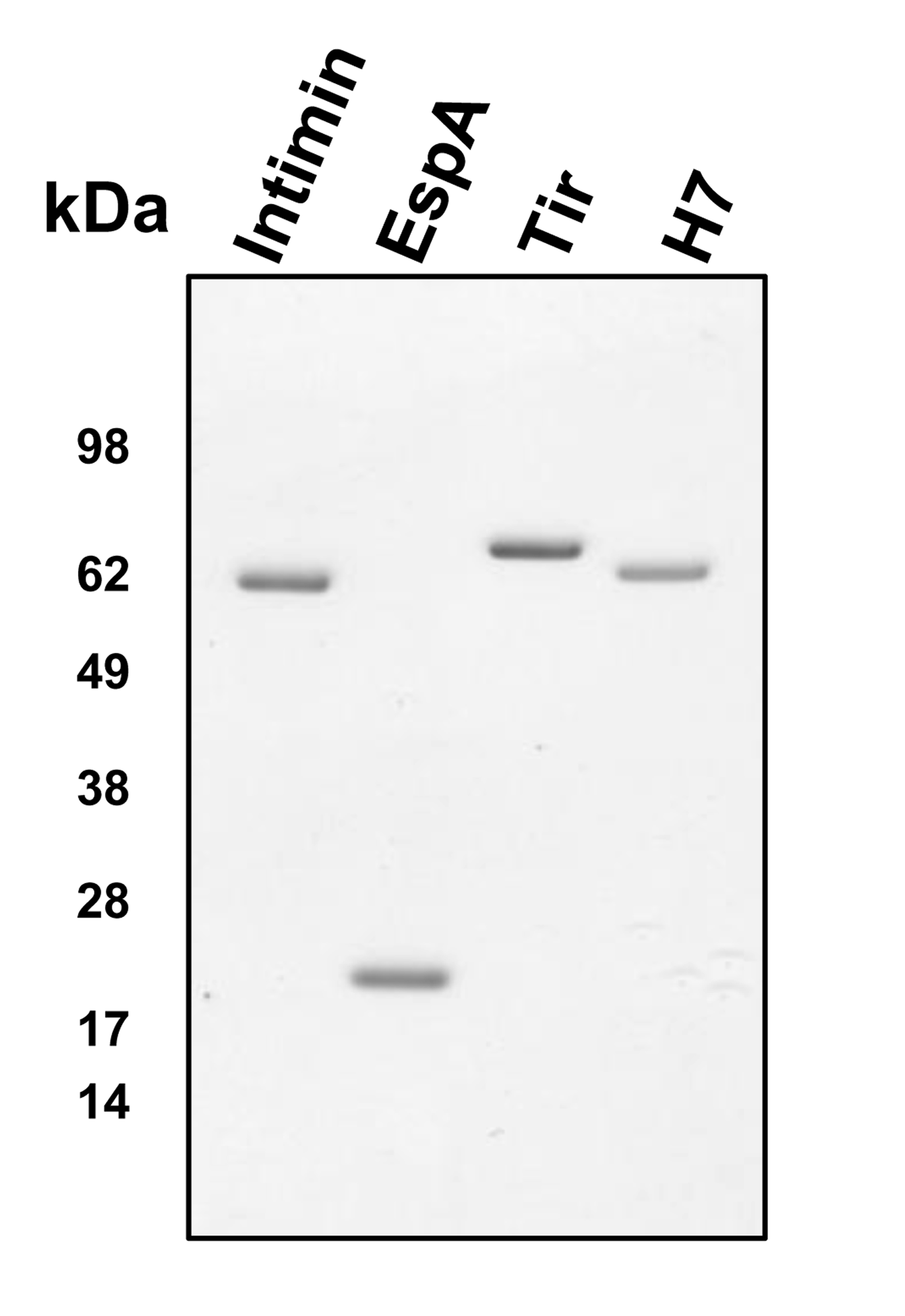

Supplement: S1 Fig — Coomassie blue-stained polyacrylamide gel of E. coli O157 antigens used in immunizations. (TIF) [file pone.0128391.s003.tif]
